# Construction of a self-luminescent cyanobacterial bioreporter that detects a broad range of bioavailable heavy metals in aquatic environments

**DOI:** 10.3389/fmicb.2015.00186

**Published:** 2015-03-09

**Authors:** Keila Martín-Betancor, Ismael Rodea-Palomares, M. A. Muñoz-Martín, Francisco Leganés, Francisca Fernández-Piñas

**Affiliations:** Department of Biology, Universidad Autónoma de MadridMadrid, Spain

**Keywords:** cyanobacteria, chemical modeling, environmental validation, free ion, heavy metal detection, self-luminescent bioreporter, *smt* locus

## Abstract

A self-luminescent bioreporter strain of the unicellular cyanobacterium *Synechococcus* sp. PCC 7942 was constructed by fusing the promoter region of the *smt* locus (encoding the transcriptional repressor SmtB and the metallothionein SmtA) to *luxCDABE* from *Photorhabdus luminescens*; the sensor *smtB* gene controlling the expression of *smtA* was cloned in the same vector. The bioreporter performance was tested with a range of heavy metals and was shown to respond linearly to divalent Zn, Cd, Cu, Co, Hg, and monovalent Ag. Chemical modeling was used to link bioreporter response with metal speciation and bioavailability. Limits of Detection (LODs), Maximum Permissive Concentrations (MPCs) and dynamic ranges for each metal were calculated in terms of free ion concentrations. The ranges of detection varied from 11 to 72 pM for Hg^2+^ (the ion to which the bioreporter was most sensitive) to 1.54–5.35 μM for Cd^2+^ with an order of decreasing sensitivity as follows: Hg^2+^ >> Cu^2+^ >> Ag^+^ > Co^2+^ ≥ Zn^2+^ > Cd^2+^. However, the maximum induction factor reached 75-fold in the case of Zn^2+^ and 56-fold in the case of Cd^2+^, implying that Zn^2+^ is the preferred metal *in vivo* for the SmtB sensor, followed by Cd^2+^, Ag^+^ and Cu^2+^ (around 45–50-fold induction), Hg^2+^ (30-fold) and finally Co^2+^ (20-fold). The bioreporter performance was tested in real environmental samples with different water matrix complexity artificially contaminated with increasing concentrations of Zn, Cd, Ag, and Cu, confirming its validity as a sensor of free heavy metal cations bioavailability in aquatic environments.

## Introduction

Heavy metals such as Cu, Ni, Fe, Zn or Co are essential for life as they are required to maintain cellular metabolism (Waldron et al., 2009; Osman and Cavet, 2010). During evolution, bacteria have developed mechanisms to sense and respond to variable fluxes of metals in the environment and try to keep a beneficial intracellular concentration of the essential metals to meet the requirements of the metalloproteins. To avoid toxicity of metals, essential or not, bacteria present detoxification and resistance systems which mainly employ proteins involved in metal efflux (CPx-ATPases, chemiosmotic efflux systems), enzymatic detoxification or intracellular sequestration of excess metal (O'Halloran, 1993; Silver and Phung, 1996; Busenlehner et al., 2003; Arguello et al., 2007; Osman and Cavet, 2010).

The ArsR-SmtB family of transcriptional repressors are a group of metal sensing transcriptional regulators which bind to proteins involved in the efflux or sequestration of metals. The family is named after the *Synechococcus* sp. PCC 7942 SmtB repressor which negatively regulates the expression of SmtA, a class II cyanobacterial metallothionein (Huckle et al., 1993; Morby et al., 1993; Turner et al., 1996; Robinson et al., 2001). *smtB* and *smtA* are divergently transcribed and repression is alleviated mainly by Zn, although it has been described that Cd, Co, Cr, Cu, Hg, Ni, and Pb increase the abundance of *smtA* transcripts (Huckle et al., 1993). Other ArsR-SmtB representatives include ZiaR which also senses Zn; CadC, Cd, Pb and Zn; CmtR, Cd and Pb; CzrA, Zn and Co; NmtR, Ni and Co; BmxR, Cu, Ag, Zn, Cd, Ni and Co and ArsR which senses As, Sb and Bi (for a review see Busenlehner et al., 2003; Osman and Cavet, 2010).

The metal sensing bacterial systems have been used for the construction of whole cell bioreporters (Hynninen and Virta, 2010). Whole cell bioreporters complement the traditional methods of detection of heavy metals which are based on highly sensitive and specific physical and chemical techniques, such as atomic absorption spectroscopy or mass spectrometry; however, such methods are not able to distinguish between available (potentially hazardous to biological systems) and non-available fractions of metals existing in the environment. In contrast to chemical methods, whole-cell bioreporters measure bioavailable metals, which is the fraction interacting with the cell and capable of passing through cellular membranes. They are also able to integrate the complexity of environmental factors (pH, redox potential, exchangeable cations, biological activity, etc.) that contribute to bioavailability (Kohler et al., 2000). In general, whole-cell bioreporters are intact living cells genetically engineered to produce a dose-dependent measurable signal in response to chemical or physical agents in their environment (Harms et al., 2006; van der Meer and Belkin, 2010). Cyanobacteria are the only prokaryotic organisms carrying out an oxygen-evolving photosynthesis. They originated during the Precambrian era, and as a group they are known to survive a wide spectrum of environmental stresses. As primary producers with a key role in the N and C cycles, they are a dominant component of marine and freshwater phytoplankton, and are well suited for detecting contaminants in aqueous samples (Bachmann, 2003; Rodea-Palomares et al., 2009). Cyanobacteria are ecologically relevant (particularly in aquatic environments), and for that reason, in recent years there has been an interest in developing recombinant bioluminescent cyanobacterial bioreporters which may be useful to assess toxicity in photosynthetic organisms (Shao et al., 2002; Rodea-Palomares et al., 2009, 2010), nutrient bioavailability due to their pivotal role in biogeochemical cycles (Bullerjahn et al., 2010; Munoz-Martin et al., 2011, 2014b) and a few specific pollutants like heavy metals (Erbe et al., 1996; Peca et al., 2008). Regarding the *smtB-smtA* locus, there is only one previous report of a cyanobacterial bioreporter based on the fusion of the complete *Vibrio fischeri luxCDABE* (now denoted as *Aliivibrio fischeri*) operon to the *smtA* promoter region of *Synechococcus* sp. PCC 7942 (Erbe et al., 1996). However, this bioreporter showed limited production of endogenous aldehyde (the luciferase substrate for the bioluminescence reaction) and needed the exogenous addition of n-decanal; it was tested with only three metals, Zn, Cu, and Cd and was shown to respond to them with varying sensitivities. In the present work, we have fused the *luxCDABE* operon from *Photorhabdus luminescens* (Szittner and Meighen, 1990; Fernandez-Pinas et al., 2000) to the promoter region of *smtA* of *Synechococcus* sp. PCC 7942 to develop a novel self-luminescent heavy-metal bioreporter which does not need the addition of exogenous aldehyde; the regulatory *smtB* gene controlling the expression of *smtA* was cloned in the same vector. We have characterized its response to a range of heavy metals: Zn, Cd, Cu, Co, Ag, Hg, Sr, Mg, Fe, Ba, Ni, and Pb. We have found that this bioreporter is induced in the presence of Zn, Cd, Cu, Co, and Hg, but interestingly also in the presence of Ag, a monovalent metal cation to which *smtA* of *Synechococcus* sp. PCC 7942 has never been reported to respond. Furthermore, due to the importance of testing the bioreporter response in real environmental samples, the bioreporter has been tested in river and wastewater samples spiked with heavy metals as case studies; as metal speciation is a relevant issue which has been seldom addressed when testing bioreporters in real samples, we have used chemical modeling (Visual MINTEQ program) in an attempt to link the bioreporter response with metal speciation and bioavailability.

## Material and methods

### Bacterial strains and culture conditions

*Synechococcus elongatus* PCC 7942 cells were grown at 28°C with continuous illumination, at 60 μmol photons m^2^s^−1^ intensity on a rotary shaker in 500 mL Erlenmeyer flasks containing 300 mL of BG11 medium (Rippka, 1988) buffered with 2 mM MOPS and pH = 7.5 (medium composition in Supplementary material Table S1). Culture medium was supplemented with 3.75 μg/mL chloramphenicol (Cm) for the transformed strain *Synechococcus elongatus* PCC 7942 pBG2120.

### Chemical substances

Enzymes required for molecular cloning were from Takara and Fermentas. Kits for plasmid extraction and purification were from Qiagen and Promega. Metal salts used in this study were: ZnCl_2_, CdCl_2_, AgSO_4_, CuSO_4_, HgCl_2_, CoCl_2_, PbNO_3_, MgCl_2_, NiCl_2_, FeCl_2_, BaCl_2_, and SrCl_2_. All metal salts were from Sigma-Aldrich. Concentrated metal salts solutions (1000 mg/L) were prepared in deionized water (Millipore) and stored at 4°C in opaque bottles. Dilutions were carried out in distilled water from this concentrated solution and kept for 1 month at 4°C.

### Construction of the smtAB-luxCDABE cyanobacterial reporter strain

**Figure 1:**
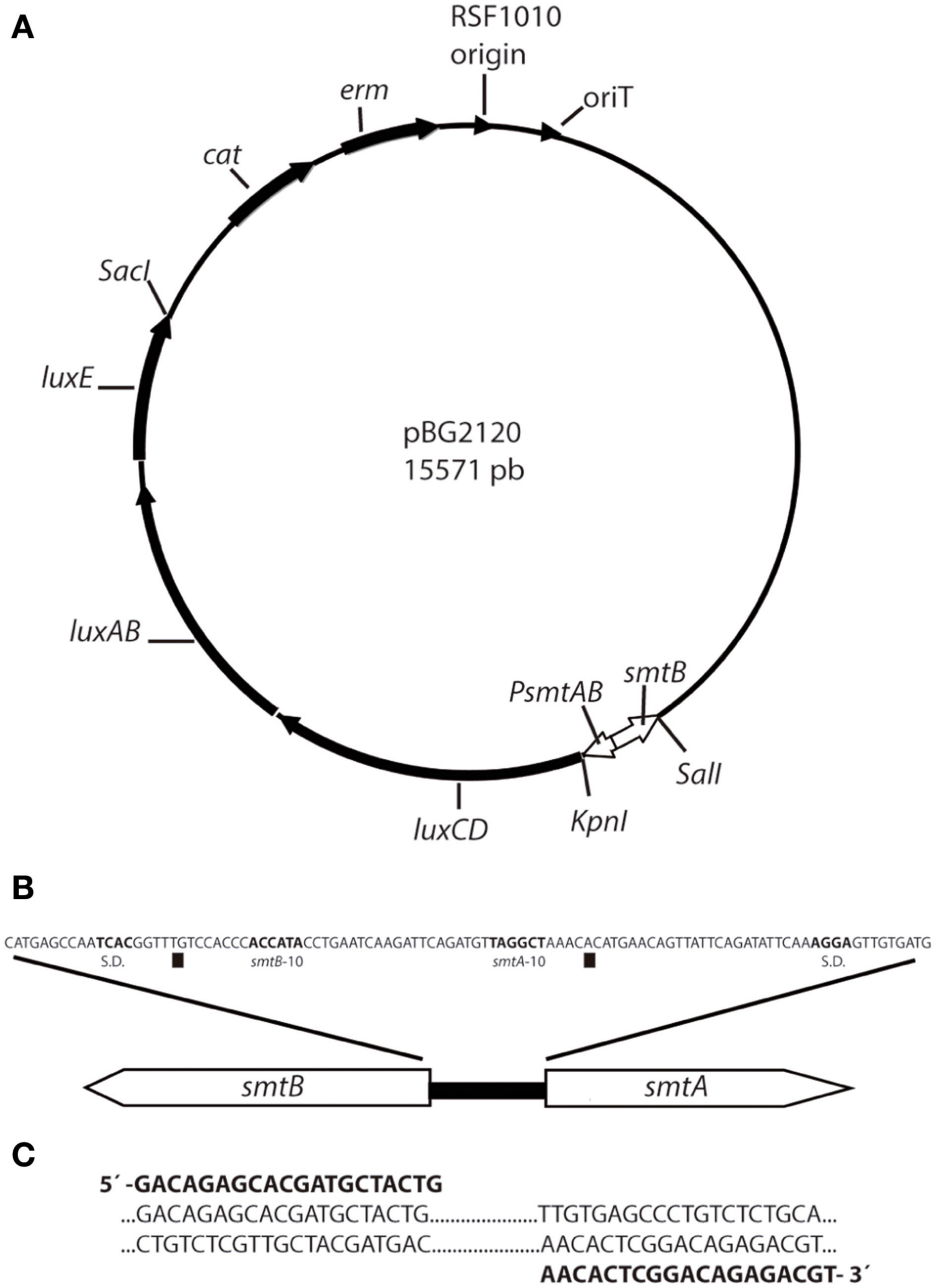
**Scheme of plasmid pBG2120 construction**. **(A)** pBG2120 resulted of cloning a sequence containing the *smtB* gene, the *smt* operator/promoter region and the first 50 pb of *smtA* in *Sal*I/*Kpn*I site of pBG2106. Transcription of the *lux* operon in pBG2120 is under the control of the *smtAB* promoter (*PsmtAB*). **(B)** Structure of the chromosomal *smt* locus of *Synechococcus* sp. PCC 7942. The operator/promoter region lies between the *smtA* and the *smtB* protein coding regions and contains divergent overlapping promoters of the two genes [squares indicate the *smtA* and *smtB* transcription start site, Shine-Dalgarno sequence (S.D.) and −10 regions are indicated in bold)]. **(C)** Sequence of PCR primers (shown in bold) used to amplify the *smt* sequence including *smtB*, the 100 bp operator/promoter region and 50 bp of *smtA*.

The cyanobacterial whole cell bioreporter for heavy metals detection was constructed using *Synechococcus elongatus* PCC 7942 as host cell. A sequence containing the *smtB* gene, the *smt* operator/promoter region and the first 50 pb of *smtA* was amplified from the genomic DNA using the primers displayed in Figure 1. The product of the PCR amplification was cloned in pDrive cloning vector (Qiagen), digested with *Kpn*I and *Sal*I and cloned into the *Kpn*I/*Sal*I sites of plasmid pBG2106 (Munoz-Martin et al., 2011) that harbors the promoterless *luxCDABE* from *Photorhabdus luminescens*, generating the plasmid pBG2120 (Figure 1). The resulting plasmid contains a *smt-luxCDABE* transcriptional fusion where the *luxCDABE* operon is regulated under the control of the metal inducible promoter region of *smtAB* and, also contains the regulatory gene *smtB*. The integrity of the construction in *Escherichia coli* (*E. coli*) was confirmed by restriction analysis and DNA sequencing.

This plasmid, pBG2120, was introduced into the cyanobacterial strain by conjugation as described (Elhai and Wolk, 1988; Elhai et al., 1997). The integrity of the transformation in *Synechococcus* sp. PCC 7942 was confirmed by PCR with the forward primer used for the amplification of the promoter and a primer designated as luxOUT, which anneals in the *luxCDABE* coding sequence: 5′-AGTCATTCAATATTGGCAGG-3′ (not included in Figure 1). A scheme depicting plasmid pBG2120 can be found in Figure 1.

### Bioluminescence assays

The bioreporter strain was grown until reaching the mid-log phase (OD_750*nm*_ = 0.6–0.7) because this growth stage was found to increase luminescence induction (data not shown) and washed twice in a BG11 modified medium lacking Co, Zn, and Cu which might induce the *smt*-*luxCDABE* reporter system (Huckle et al., 1993) (Supplementary Material Table S1) buffered with 2 mM MOPS and pH = 7.5. For standardization purposes cells were resuspended in the same medium to reach a final OD_750*nm*_ = 0.5 (Rodea-Palomares et al., 2009). Metal salt exposure experiments were performed in transparent 24 well microtiter plates in a 1.5 mL final volume. Metal salts were added to the wells to get the desired final concentrations, which were between 0 and 40 μM for each tested metal.

Plates were incubated at 28°C in light (60 μmol m^2^s^−1^) on a rotatory shaker up to 6 h. For the luminescence measurements, 100 μl of cell suspensions were transferred to an opaque 96-well microtiter plate and luminescence was recorded every 5 min for 20 min in a Centro LB 960 luminometer (Berthold Technologies GmbH and Co.KG, Bald Wilbad, Germany) and the maximum record (usually at 15–17 min) was taken. All data are expressed as Bioluminescence induction factors (BIFs) calculated by dividing the mean luminescence signal of a treated sample by the mean luminescence signal of the untreated sample. The limits of detection (LODs) were defined as a value two-fold above the background signal plus three times the standard deviation. In addition, the maximum permissive concentrations (MPCs), the highest concentrations that do not cause toxicity to an organism, were determined. The luminescence was measured without supplementation of exogenous aldehyde as the strain harbored the *lux* operon and its endogenously generated aldehyde was not limiting (as tested by the addition of exogenous aldehyde, data not shown).

### Toxicity bioassay

The toxicity of the heavy metals was determined by monitoring growth inhibition of the cyanobacterium *Synechococcus* sp. PCC 7942 pBG2120. Strain manipulation and metal tested concentrations were the same as described for bioluminescence bioassays. The bioreporter strain was grown until reaching the mid-log phase (OD_750*nm*_ = 0.6–0.7), washed twice in a BG11 modified medium lacking Co, Zn, and Cu buffered with 2 mM MOPS and pH = 7.5 and resuspended in the same medium to reach a final OD_750*nm*_ = 0.5. Experiments were performed in 24 well microtiter plates in a 1.5 mL final volume. Metals were added to the wells to get the desired final concentrations. The growth of *Synechococcus* sp. PCC 7942 pBG2120 was monitored for 4 and 20 h and assessed by optical density at 750 nm using a HITACHI U-2000 spectrophotometer. Microplates were maintained at 28°C inside a growing chamber with controlled light intensity (60 μmol photons m^2^s^−1^) on an orbital shaker. Three independent experiments with triplicate samples were conducted.

### Spiking experiments: artificial contamination of environmental water samples with heavy metals

Three environmental water samples with different matrix compositions were selected to validate the response of the metal reporter under real environmental conditions, as explained below:

Two fresh water samples were from the Guadalix River (Glx); this is a tributary of the larger Jarama River and is located in central Spain, near the city of Madrid. It is 38 km long and flows mainly through siliceous substrates (Douterelo et al., 2004). The main flow is about 1 m^3^/s after the thaw and no more than 0.60 m^3^/s the rest of the year. The source of the river is at more than 1790 m altitude in the south drainage Guadarrama Mountains and flows into the Jarama River at 600 m altitude. Glx1 sampling point is located near the headwaters at 1500 m and does not have anthropogenic influence, whereas the Glx3 sampling point is near a human settlement, San Agustín de Guadalix. At this point, the river receives industrial and domestic sewage.

Moreover, a wastewater sample was collected from the effluent of the secondary clarifier of the Alcalá de Henares wastewater treatment plant (WWTP) (Rosal et al., 2010; Barran-Berdon et al., 2011). This is the main WWTP in the region and it discharges treated wastewater effluents into the Henares River. The location of the sampling points are shown in Supplementary Material Figure S1. The water sample manipulation, storage and analysis were performed essentially as previously described (Rodea-Palomares et al., 2010; Munoz-Martin et al., 2011). Quantitative analysis of the elemental composition of the waters samples was performed by inductively coupled plasma–mass spectrometry (ICP–MS; Perkin–Elmer Sciex Elan 6000 equipped with an AS 91 autosampler) by the ICP–MS laboratory of the Universidad Autonoma de Madrid. The main environmental water physicochemical characteristics are described in Table 1. For the heavy metal spiking experiments, 75 μl of the concentrated culture was added to the environmental water samples, already pre-incubated with metals for at least 30 min (Fernandez-Pinas et al., 1991), to reach a final OD_*750nm*_ of 0.5 in a final volume of 1.5 mL. The water samples were supplemented with BG11 (without Cu, Zn and Co, as described above) growth medium, to ensure that any change in luminescence was not due to any nutrient deficiency; 150 μl ten-fold concentrated medium was added so that, in the final volume of 1.5 mL, the composition of ions in the supplemented samples was the same as that in BG11; dilution of the sample by addition of BG11 did not significantly change Visual MINTEQ predictions. The metals and the range of nominal concentration tested were as follows: ZnCl_2_: [3.6–15 μM], CdCl_2_: [2.45–10 μM], AgSO_4_: [0.2–0.5 μM], and CuSO_4_: [0.875–7 μM]. Plates were incubated at 28°C in light (60 μmol m^2^s^−1^) on a rotatory shaker for 4 h (see Results). For the luminescence measurements, 100 μl of cell suspensions were transferred to an opaque 96-well microtiter plate and recorded every 5 min for 20 min in a Centro LB 960 luminometer (Berthold Technologies GmbH and Co.KG, Bald Wilbad, Germany) and the maximum record (usually at 15–17 min) was taken. All data are expressed as BIFs calculated by dividing the mean luminescence signal of a treated sample by the mean luminescence signal of the untreated sample. Triplicate samples within each experiment were measured in at least three independent experiments.

**Table 1:** **Main physicochemical characteristics of environmental waters used in the study**.

**Physicochemical parameters**	**Guadalix river**		**Alcalá wastewater treatment plant (WWTP)**
	**Glx1**	**Glx3**	
Water temperature (°C)	8.6	9.9	13
pH	6.9	7.2	7.5
Conductivity (μs cm^−1^)	100	325	702
PO^3−4^ –P (mg l^−1^)	0.05	0.24	1.1
Alkalinity (mg l^−1^ CaCO_3_)	14.5	80	472
Hardness (mg l^−1^ CaCO_3_)	17.7	109	176
N-NO^−^_3_ (mg l^−1^)	0.2	0.45	7
N-NH^+^_4_ (mg l^−1^)	0.05	0.14	1.5
Microelements (μM)			
Mg	67.64	336.4	786.07
Na	398.78	737.29	3575.92
K	21.20	90.63	447.54
Ca	24.95	986.18	1117.85
Mn	0.027	0.024	0.49
Fe	0.19	0.081	1.05
Co	6.79·10^−4^	1.02·10^−3^	0.012
Ni	5.28·10^−3^	8.004·10^−3^	0.095
Cu	3.51·10^−3^	6.41·10^−3^	0.036
Zn	0.74	0.43	0
As	0.065	0.2	0.057
Ag	0	2.78·10^−4^	8.84·10^−3^
Cd	0	0	0
Hg	0	0	2.48·10^−3^
Pb	0	0	0.01
Sr	0.47	3.34	10.07
Ba	0.12	0.13	0.05

*Glx1, Guadalix river sampling point as a representative of the upstream course, Glx3 as representative of the downstream course; WWTP, effluent of Alcalá de Henares Wastewater Treatment Plant*.

### Modeling of metal speciation

The chemical equilibrium model Visual MINTEQ (http://www.lwr.kth.se/English/OurSoftware/vminteq/index.htm) was used to predict the metal speciation with the growth medium (BG11) and environmental samples. Assumptions of a fixed pH, fixed potential redox (Eh), closed system and no precipitation of solid phases were made during computations. This chemical model has proved very useful for linking speciation to metal toxicity and biosorption processes in a number of organisms (Newman and McCloskey, 1996; Campbell et al., 2000; Deheyn et al., 2004; Herrero et al., 2005; Rodea-Palomares et al., 2009). In the present work, Visual MINTEQ is used in order to predict metal speciation and link it to the response of the reporter strain in the assay and in the spiking experiments. The LODs, dynamic ranges and MPCs of the bioreporter performance given in the text were calculated based on the free ion metal concentration as predicted by Visual MINTEQ for the assay and spiking experiments.

### Statistical analysis

The test of statistically significant differences between data sets was performed using One-Way Analyses of Variance (ANOVA); also, to discriminate which data sets were significantly different from the others, the *post-hoc* Tukey's HSD (honestly significant difference) test was performed. All the tests were computed using R software 3.0.2. (copyright©The Foundation for Statistical Computing). All data were obtained from a minimum of three independent experiments with replicates for each assay condition. Toxicity was expressed as effective concentration EC_50_ which is the metal concentration exerting 50% growth inhibition. To calculate the EC_50_ values, dose-response curves were fitted by non-linear parametric functions with the R “*drc*” analysis package (Ritz and Streibig, 2005) (R for windows, 3.0.2 version Development Core Team). Best-fit models were selected by using the “model select” function provided in the *drc* package according to the maximum likelihood and the Akaike's information criterion (Ritz and Streibig, 2005).

## Results

### Selectivity and sensitivity of *Synechococcus* sp. PCC 7942 pBG2120 to heavy metals

**Figure 2:**
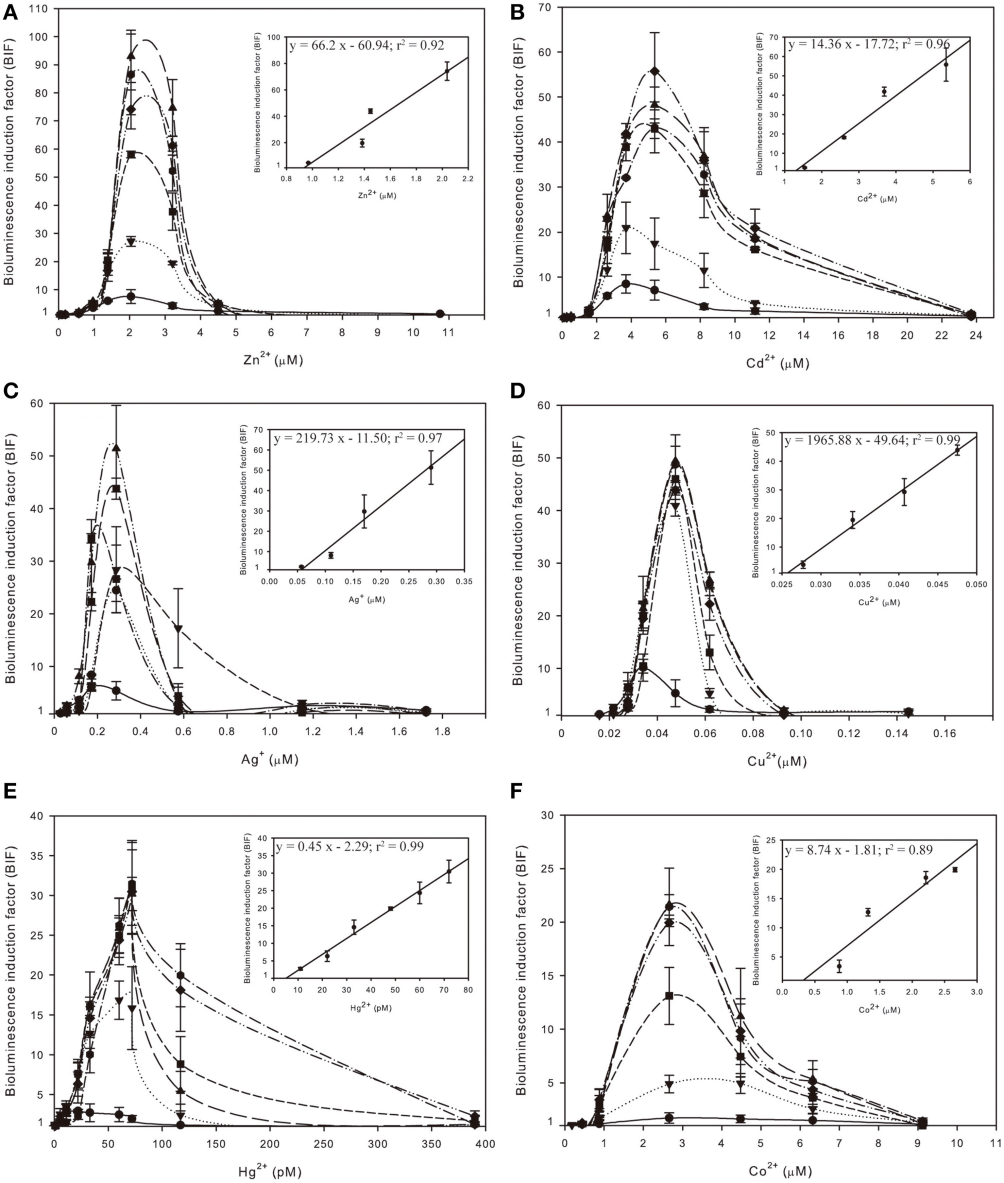
**Inducibility of *Synechococcus* sp. PCC 7942 pBG2120 based on free ion metal concentration as predicted by Visual MINTEQ to the metals salts: ZnCl_2_ (A), CdCl_2_ (B), AgSO_4_ (C), CuSO_4_ (D), HgCl_2_ (E) and CoCl_2_ (F) during 1–6 h exposure times: 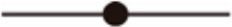 (1 h); 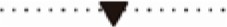 (2 h); 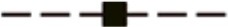 (3 h); 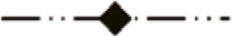 (4 h); 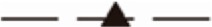 (5 h) and 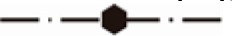 (6 h)**. Each figure contains an inset with the regression curve for each free ion metal after 4 h of exposure time. Data represent the mean ± standard deviation of at least three independent experiments.

The selectivity and sensitivity profile of *Synechococcus* sp. PCC 7942 pBG2120 was studied as a function of exposure time to twelve different metals: Zn, Cd, Ag, Cu, Hg, Co, Pb, Mg, Ni, Fe, Ba, and Sr. Six metals effectively induced the *smtAB::luxCDABE* reporter system of *Synechococcus* sp. PCC 7942 pBG2120: Zn, Cd, Ag, Cu, Hg, and Co. Their induction profiles for a range of metal concentrations expressed as free ion, and exposure times are shown in Figure 2. No significant induction of the *smtAB::luxCDABE* reporter system was observed for any of the other six metals tested: Pb, Mg, Ni, Fe, Ba, and Sr (Supplementary Material Figure S2).

The medium used for the assays was BG11. This is the growth medium for this organism, and it had to be used also as bioassay medium (without trace metals) because in water the bioreporter strain was not induced to the same level as when assay was performed in growth medium. Because the medium used for the assays, BG11 (medium composition in Supplementary Material Table S1), is a rich medium and it has components that can complex heavy metals, such as phosphate, EDTA and ferric ammonium citrate, the chemical program Visual MINTEQ was used to predict metal speciation. The predicted percentages of metal present as free ion in BG11 medium and the other main chemical species in BG11 medium are detailed in Supplementary Material Table S2. The BIFs shown in Figure 2 were calculated based on free ion metal concentration as predicted by Visual MINTEQ.

As shown in Figure 2, for each metal, BIF values increased in a dose-dependent fashion as a function of exposure time up to 4 h. Longer exposure times did not result in a significant higher induction. The maximum BIF for Zn^2+^ (Figure 2A) was the highest, near 75-fold induction followed by Cd^2+^ (Figure 2B) with a maximum BIF of 56-fold induction, Ag^+^ (Figure 2C) with a maximum BIF of 50-fold induction, Cu^2+^ (Figure 2D) with a maximum BIF of 45-fold induction, Hg^2+^ (Figure 2E) with a maximum BIF of 30-fold induction and Co^2+^ (Figure 2F) with a maximum BIF of 20-fold induction.

The LODs, MPCs and dynamic ranges of performance of *Synechococcus* sp. PCC 7942 pBG2120 for each metal in terms of free ion are summarized in Table 2.

**Table 2:** **Limits of detection (LODs), free ion dynamic ranges, free ion maximum permissive concentrations (MPCs), regression equations and corresponding *R*^2^ values for Synechococcus sp. PCC 7942 pBG2120 for each metal tested in terms of free ion**.

**Metal**	**Free ion LODs (μM)**	**Free ion dynamic range (μM)**	**Free ion MPCs (μM)**	**Regression equation**	***R*^2^**
Zn^2+^	0.97	0.97–2.04	2.04	*y* = 66.2 *x* – 60.94	0.92
Cd^2+^	1.54	1.54–5.35	5.35	*y* = 14.36 *x* – 17.72	0.96
Ag^+^	0.05	0.05–0.29	0.29	*y* = 219.73 *x* – 11.50	0.97
Cu^2+^	0.027	0.027–0.05	0.05	*y* = 1965.88 *x* – 49.64	0.99
Hg^2+^	11^*^	11–72^*^	72^*^	*y* = 0.45 *x* – 2.29	0.99
Co^2+^	0.88	0.88–2.66	2.66	*y* = 8.74 *x* – 1.81	0.89

* *, Hg^2^+ concentrations are in picomolar (pM); y = bioluminescence induction factor (BIF); x = free ion metal concentration (μM)*.

Regarding sensitivity, the lowest LOD was found for Hg^2+^, followed in decreasing order of sensitivity by Cu^2+^, Ag^+^, Co^2+^, Zn^2+^, and Cd^2+^. This order of sensitivity was further confirmed by a toxicity bioassay using growth inhibition as the endpoint after 20 h exposure to increasing concentrations of metals (see Supplementary Material Table S3 with EC_50_ values; exposure times shorter than 20 h did not allow calculations of EC_50_ values). The observed luminescence decrease, abrupt in many of the cases, at concentrations higher than the MPCs indicated metal toxicity as shown by the quite low EC_50_ values obtained in the toxicity bioassays.

Table 2 also shows the main parameters of the regression curves in the linear ranges of the bioreporter response; further experiments including intermediate concentrations were carried out to confirm the linearity range for each metal; the regression curves were plotted as a fraction of free ion in the exposure medium using chemical modeling (Visual MINTEQ).

### Application of *Synechococcus* sp. PCC 7942 pBG2120 as a bioreporter of heavy metal bioavailability in environmental samples: spiking experiments

The above experiments indicate that *Synechococcus* sp. PCC 7942 pBG2120 responds in a linear manner to 6 different metals in different detection ranges: Zn^2+^ (0.97–2.04 μM), Cd^2+^ (1.54–5.35 μM), Ag^+^ (0.05–0.29 μM), Cu^2+^ (0.027–0.05 μM), Hg^2+^ (11–72 pM), and Co^2+^ (0.88–2.66 μM). As described before, 4 h was found to be an optimal exposure time for all metals; therefore, environmental assays were performed after 4 h of exposure.

To test the suitability of *Synechococcus* sp. PCC 7942 pBG2120 as a bioreporter of heavy metals in environmental samples, we exposed it to three different water samples. Two samples points were from upstream and downstream of Guadalix River, Glx1 and Glx3, respectively, and the third was from an effluent of the Alcalá WWTP. These samples were artificially contaminated (spiked or doped) with increasing concentrations of Zn, Cd, Ag, and Cu as described in Material and Methods section. All sampling points are located in central Spain (Supplementary Material Figure S1) and the main environmental water physicochemical characteristics are described in the Material and Methods section and Table 1.

As can be seen in Table 1, there is a wide range of variation in the concentrations of the different parameters between the three water samples. Mainly, Glx1 represents a near pristine water with low electrical conductivity, PO^3−^_4_–P, alkalinity, N-NO^−^_3_ and N-NH^+^_4_, while Glx3 and WWTP present increasing anthropic influence represented by increasing values for those parameters. Also, Table 1 details the chemically detected concentrations of different metals present in the water samples. The concentration of the heavy metals present in the water samples were very low and below the limit of detection of the strain; in fact, the water samples did not induce the bioreporter response (data not shown).

Table 3 shows the heavy metal nominal concentrations used to spike the water samples, the predicted free ion metal concentration as predicted by Visual MINTEQ and the bioreporter output as calculated from the calibration curve given in the text, for each metal concentration in each water sample. It is very interesting to note that the predicted free ion metal concentrations were slightly lower in river sample Glx3 and considerably lower in wastewater sample for all metals added, indicating the complexity of the water matrix in the WWTP.

**Table 3:** **Heavy metal concentrations tested in water samples in the spiking experiments, predicted free ion heavy metal concentrations by Visual MINTEQ and the bioreporter output (calculated from the calibration curve given in the text) in water samples for two sampling points in Guadalix river (Glx1 as representative of the upstream course and Glx3 as representative of the downstream course) and one sample from the effluent of Alcalá de Henares wastewater treatment plant (WWTP)**.

		**Sample**					
		**Glx1**		**Glx3**		**WWTP**	
**METAL**	**Nominal Conc. (μM)**	**Predicted free ion (μM)**	**Bioreporter output (μM)**	**Predicted free ion (μM)**	**Bioreporter output (μM)**	**Predicted free ion (μM)**	**Bioreporter output (μM)**
Zn	3.6	0.86	0.96±0.003	0.77	−	0.48	−
	7.3	1.82	0.97±0.01	1.62	0.96±0.01	0.99	0.97±0.01
	10	2.57	−	2.30	−	1.37	1.14±0.12
	15	4.09	−	3.63	−	2.08	1.58±0.33
Cd	2.5	1.51	1.31±0.009	1.38	1.31±0.01	0.89	1.31±0.009
	5	3.04	1.67±0.13	2.77	1.88±0.51	1.88	1.65±0.26
	7	4.28	2.54±0.46	3.89	2.79±0.23	2.71	2.58±0.88
	10	6.14	−	5.60	−	4	4.17±0.45
Ag	0.2	0.10	0.073±0.002	0.10	0.068±0.003	0.03	−
	0.3	0.15	0.09±0.006	0.15	0.083±0.002	0.04	−
	0.5	0.25	0.157±0.002	0.26	0.164±0.002	0.07	0.165±0.012
	1	0.51	−	0.51	−	0.14	0.17±0.01
Cu	1.75	0.03	0.027±0.001	0.03	0.027±0.001	0.022	−
	3.5	0.07	0.036±0.005	0.06	0.042±0.007	0.022	−
	7	0.16	−	0.13	−	0.05	0.03±0.0006
	14	0.37	−	0.28	−	0.13	0.039±0.005

*The bioreporter output data represent the average ± standards errors of the mean (n = 3)*.

*−, out of calibration range*.

As can be seen in Table 3, in the case of Zn^2+^, the metal concentration estimated based on the bioreporter output was 53.3% of that predicted by Visual MINTEQ in Glx1 and 60% in Glx3. In the case of Cd^2+^, the metal concentration estimated based on the bioreporter output was an average of 57% of that predicted by Visual MINTEQ in Glx1 and 70% in Glx3. In the WWTP sample, the metal concentration estimated based on the bioreporter output was 86% and 96% of that predicted by Visual MINTEQ for Zn^2+^ and Cd^2+^, respectively. In the case of Ag^+^, the metal concentration estimated based on the bioreporter output was 65% of that predicted by Visual MINTEQ in Glx1 and 62% in Glx3.

Interestingly, in the WWTP, the amount of Ag^+^ detected by *Synechococcus* sp. PCC 7942 pBG2120 in the water sample with metal concentration within its dynamic range, was higher than that predicted by Visual MINTEQ (around 2-fold).

In the case of Cu^2+^, the metal concentration estimated based on the bioreporter output was 70% and 80% of the concentration predicted by Visual MINTEQ in both river samples, Glx1 and Glx3, respectively; in the WWTP, only the highest concentrations tested was within the calibration range for this metal and the concentration estimated based on the bioreporter output was 60% of that predicted by Visual MINTEQ.

For some of the spiked samples, the obtained output was out of the calibration range of the bioreporter; in those with metal concentration above the calculated MPCs, the unreliability of the bioreporter was probably due to toxicity (Supplementary Material Table S3).

**Figure 3:**
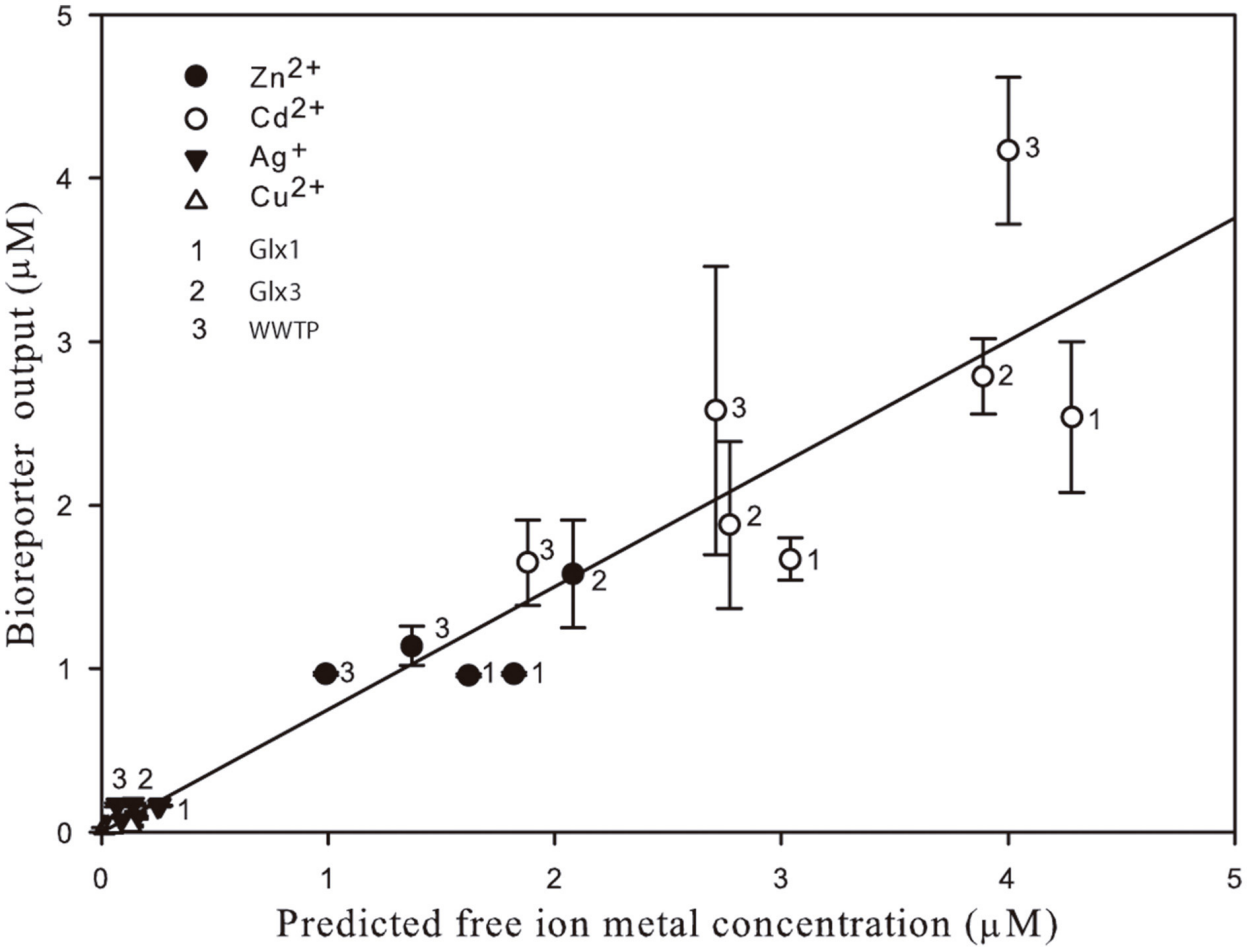
**Free ion metal concentrations as predicted by Visual MINTEQ *vs*. bioreporter output for all the heavy metal tested (Zn^2+^, Cd^2+^, Ag^+^, and Cu^2+^) detected by *Synechococcus* sp. PCC 7942 pBG2120 in all the environmental matrices used in the environmental waters' spiking experiments**.

Figure 3 shows free ion concentrations as predicted by Visual MINTEQ *vs*. bioreporter output for all the heavy metal tested and all the environmental matrices used as a summary of the global performance of the *Synechococcus* sp. PCC 7942 pBG2120 estimating bioavailable heavy metals in environmental water matrices. As can be seen in the figure, within its dynamic ranges, the global performance of this bioreporter strain is excellent and, in general, it was very accurate for all metals tested in all water samples. A global high linear correlation (*r*^2^ = 0.9) free ion predicted by Visual MINTEQ *vs*. bioreporter output was obtained.

## Discussion

In this study, we report the construction, characterization and testing in actual environmental samples of a self-luminescent cyanobacterial bioreporter sensitive to several heavy metals both essential and non-essential for life. The constructed *Synechococcus* bioreporter is based on the *smt* locus present in the same cyanobacterium. The *smt* locus consists of a regulatory gene, *smtB*, which depending on the bioavailability of certain metals, controls the expression of *smtA*. This gene encodes a metallothionein whose main role is to sequester an excess of the heavy metal ions in the bacterial cell. This is a well-known system of bacterial metal sensing proteins (see Osman and Cavet, 2010 for a thorough review) and may be useful to construct bacterial bioreporters to detect heavy metals. In fact, Erbe et al. (1996) already constructed what can be considered the first cyanobacterial bioreporter able to detect heavy metals; it was also based on the *smt* locus and the host was also *Synechococcus* PCC 7942. However, the genetic construct was different as they fused the *smt* operator/promoter region to *A. fischeri luxCDABE* operon. The resulting recombinant strain was not able to produce enough endogenous aldehyde (the substrate for luciferase), therefore exogenous aldehyde had to be added for the bioluminescent reaction to occur. In contrast, the bioreporter that we have constructed is a fusion of the *smt* operator/promoter region with *Photorhabdus luminescens luxCDABE*, which rendered the strain self-luminescent with no need of exogenous aldehyde. The addition of aldehyde increased bioluminescence but did not modify the dynamic ranges of detection by the bioreporter. Furthermore, this bacterial luciferase has the greatest thermal stability among bacterial luciferases (Szittner and Meighen, 1990; Fernandez-Pinas et al., 2000) and allows monitoring at higher temperatures than that of *A. fischeri*. This fact helps to decrease the toxicity caused by the addition of exogenous aldehyde (Fernandez-Pinas et al., 2000; Porta et al., 2003).

Erbe et al. (1996) tested the sensitivity and specificity of the bioreporter toward three metal salts: ZnCl_2_, CuSO_4_, and CdCl_2_. Although calibration curves, LODs or dynamic ranges for detection were not provided, the authors found a linear increase of luminescence with increasing ZnCl_2_ from 0.5 to 2 μM after 1 h of exposure with 5 μM decreasing luminescence. Regarding CuSO_4_ and CdCl_2_, they found that greater concentrations than 15 μM CuSO_4_ and 1.5 μM CdCl_2_ reduced luminescence due to toxicity. To our knowledge this bioreporter has not been tested with environmental samples. Regarding our bioreporter, growth of cells and assays were performed in BG11 growth medium (as Erbe et al., 1996) without metal salts added but retaining all the other components that were necessary for growth and induction of the bioreporter. BG11 medium has components such as phosphate, citrate or EDTA which may precipitate/chelate the metal ions and for this reason, chemical modeling (Visual MINTEQ) (Rodea-Palomares et al., 2009) was used to link metal speciation with the bioreporter response. We have calculated the calibration curves and subsequent LODs and dynamic ranges as a function of the free ion which is usually the most bioavailable and toxic metal species (Sunda and Lewis, 1978). This approach is not currently taken when calculating the dynamic ranges of heavy metal bioreporters (Magrisso et al., 2008) except for cyanobacterial iron bioreporters (Durham et al., 2002; Boyanapalli et al., 2007). The dynamic ranges calculated using this method show that the constructed cyanobacterial bioreporter varies in sensitivity with the different metals tested. While Sr, Mg, Fe, Ba, Ni, and Pb did not induce the bioreporter luminescence, the rest of the metals ranges of detection varied from 11 to 72 pM for Hg^2+^ (the ion to which the bioreporter was most sensitive) to 1.54–5.35 μM for Cd^2+^ with an order of decreasing sensitivity as follows: Hg^2+^ >> Cu^2+^ >> Ag^+^ > Co^2+^ ≥ Zn^2+^ > Cd^2+^. This order of decreasing sensitivity was further confirmed by the toxicity bioassay. In general, non-essential ions are detected at much lower concentrations than the essential ones because they are not needed and are usually more toxic (Hynninen and Virta, 2010). However, the sensitivity of bacterial bioreporters also rely on a combination of metal affinities by the sensors and homeostasis/resistance mechanisms that determines the intracellular concentration available for detection. In this regard, if a metal is actively excluded by the cell, its cytoplasmic pool may not reach a threshold level needed for detection by a particular sensor (Cavet et al., 2003). Zn, Cu, and Co are essential for cyanobacteria (Cavet et al., 2003) while Hg, Ag and Cd are not. The fact that Cu, although essential, is the second in order of decreasing sensitivity could be due to the elevated toxicity of this element to the bioreporter as found in the toxicity bioassay. Cu has been reported to adversely affect phytoplankton, inhibiting photosynthesis and causing serious cell damage through the formation of reactive oxygen species (Wei et al., 2014). From the tested metals, Cd was the least toxic and the bioreporter showed the smallest sensitivity to this ion. Cd is a Zn analog which enters bacterial cells via transport systems for essential divalent cations and, it is known, in many bacteria, that cellular extrusion mechanisms do not usually differentiate between both ions (Hynninen and Virta, 2010). That might be a plausible explanation for the observed similar sensitivity for both Cd and Zn by the SmtB sensor.

The constructed bioreporter shows a broad specificity as it responds, although with varying sensitivities, to Zn^2+^, Cd^2+^, Co^2+^, Cu^2+^, Hg^2+^, and Ag^+^. Huckle et al. (1993) already demonstrated *in vitro* that Cd, Co, Cr, Cu, Hg, Ni, and Pb increased the expression of *smtA*. By fusing the upstream region of *smtA* to the reporter gene *lacZ*, they found that, *in vivo*, Zn was the most effective elicitor of *smtA* expression followed by Cu, Cd, Co, and Ni. *In vivo*, we did not get any induction by Pb or Ni; regarding Co, Cavet et al. (2002) using the same cyanobacterial host fount that *in vitro* SmtB binds to Co^2+^ but *in vivo*, also using a *lacZ* reporter fusion, Co^2+^ did not induce *smtA* in *Synechococcus*. It is interesting that our bioreporter responded *in vivo* to a monovalent cation, Ag^+^, which had not been found to induce this system before. Nevertheless in the filamentous cyanobacterium *Oscillatoria brevis*, Liu et al. (2004) found a *smtB* ortholog, denoted as *bxmR* encoding a regulatory protein BmxR which has been found to respond both to mono (Ag^+^; Cu^+^) and divalent (Cd^2+^, Zn^2+^) metal cations. However, although regarding sensitivity the bioreporter senses Zn^2+^ and Cd^2+^ at concentrations higher than those of other metal ions, it should be pointed out that the maximum induction factor reaches 75-fold in the case of Zn^2+^ and 56-fold in the case of Cd^2+^, implying that as found by Huckle et al. (1993) Zn^2+^ is the preferred metal *in vivo* for SmtB, followed by Cd^2+^, Ag^+^ and Cu^2+^ (around 45–50-fold induction), Hg^2+^ (30-fold), and finally Co^2+^ (20-fold).

SmtB is an intracellular metal sensor. We do not know which intracellular metal species are sensed by SmtB, however, the free ion could be a good candidate. It has been found that in the case of Zn, free Zn^2+^ is the major form of this nutrient taken up by the phytoplankton (Barnett et al., 2012). Once inside the cells, the intracellular metal pools might vary depending on metalloproteins that may bind essential metal ions as cofactors, metallothioneins which may sequester ions (essential or not) in excess or unknown intracellular ligands which might also chelate the free ions. The metal sensors regulate the abundance of their cognate metal handling protein but, also, there are efflux pumps to provide ion homeostasis both for essential and non-essential ions (Rae et al., 1999; Blindauer, 2008; Choi and Bird, 2014). From studies based on intracellular Cu^2+^ and Zn^2+^ homeostasis, it is known that the high chelation capacity of cells and that both ions' concentrations are tightly controlled. In yeast, it has been found that there is a great cellular capacity for Cu binding, meaning a low availability of Cu^2+^ inside the cells (less than 10^−18^ M in unstressed cells), so, it is extraordinarily restricted (Rae et al., 1999). Regarding Zn^2+^, it has been found to be tightly controlled as it has been suggested to act as a signaling element (Choi and Bird, 2014). Although the total cellular Zn quota of *E. coli* is in the micromolar range, *E. coli* was found to sense Zn^2+^ *in vivo* in the nanomolar range and *in vitro* in the femtomolar range (Outten and O'Halloran, 2001; Blindauer, 2008). This difference between *in vitro* and *in vivo* sensing has been attributed to ligands in the cytosol that may play an important role in Zn^2+^ buffering. As suggested by Cavet et al. (2002), there is a need in cyanobacteria and other cell types to identify the chemical form(s) of the labile pool(s) of metals accessible by each metal sensor to understand the observed sensitivities and requirements of metalloproteins, which is essential information to understand the response *in vivo* of the constructed metal bioreporters.

Our bioreporter response has been also tested with actual environmental samples spiked with increasing concentrations of heavy metals. The real samples used reflect water matrices of different complexity (affected or not by anthropogenic influence). This approach is seldom used in the field of bacterial bioreporters (Magrisso et al., 2008; Hynninen and Virta, 2010) although, in the case of cyanobacteria, it has been done for iron bioreporters (Durham et al., 2002; Boyanapalli et al., 2007); P and N bioreporters (Munoz-Martin et al., 2011, 2014a,b) and for Co, Zn, and Ni bioreporters constructed by Peca et al. (2008), using the *coa* and *nrs* detection systems. The concentrations of free ions detected by the bioreporter in the two river spiked samples represented an average of 68% of that predicted by chemical modeling and nearly 100% of that predicted in the spiked WWTP, which may be interpreted as the bioavailable fractions. As discussed earlier, the SmtB sensor only detects the metal ions which accumulate into the cell (the cytoplasmic pool) and this depends on the ion homeostasis of each cell system (i.e., metal exclusion systems or buffer systems such as metallothioneins) (Osman and Cavet, 2010). Besides, a fraction of the ions might not enter the cell and be adsorbed to negatively charged groups of the cell wall. In the case of Ag^+^, the bioavailable Ag^+^ as detected by the bioreporter was higher (almost double) than that predicted by chemical modeling, which probably means that all free Ag^+^ is bioavailable, but also might indicate, that Visual MINTEQ prediction is underestimating the concentration of this particular free ion, probably due to the complexity of the water matrix of the WWTP. Although the main physicochemical parameters and metal concentrations of this sample were characterized, probably has other uncharacterized components such as dissolved organic matter, unknown to us, which were not included as inputs in the chemical model. In some of the spiked environmental samples, the bioreporter did not give a reliable response as the amount of metal was higher than its MPCs. This means that in highly polluted samples, toxicity might be an issue and false negative results could be found. This drawback could be solved by serial dilution of the sample to decrease toxicity and restore induced responses (Amaro et al., 2011). To detect sample toxicity, it could also be convenient to use, in parallel with the metal bioreporter, a general toxicity bioreporter such as the cyanobacterial bioreporter *Anabaena* CPB4337 (Rodea-Palomares et al., 2009).

In Supplementary Material Table S4, we compare the sensitivity and dynamic ranges of our bioreporter strain with those of several microbial bioreporters which respond to the same metals (see Supplementary Material Table S4 for references). It is not an easy comparison since the sensing elements are quite different; we have used the promoter of a metallothionein locus while most prokaryotic bioreporters are based on promoters of metal transport systems with sensors displaying different affinities. In the table, we have included eukaryotic bioreporters (based on the ciliate *Tetrahymena termophila*) which are also based on metallothionein promoters (Amaro et al., 2011). There are also differences in the luciferase systems used in the bioreporters since, although many are based on bacterial luciferases, others are based on the eukaryotic firefly luciferase and the two reporter systems differ in sensitivity. Also, as indicated in Supplementary Material Table S4, different assay media have been used and usually no prediction/calculation of particular metal species has been made. Taking into account these limitations, the cyanobacterial bioreporter constructed in this study is, in general, more sensitive (based in the published LODs) to Hg, Cu, and Ag than other microbial bioreporters. It is less sensitive to Co than the *coaT*-based *Synechocystis* bioreporter. Regarding Zn, it shows a similar sensitivity to that of the ciliate bioreporter and the previous *smtAB*-based cyanobacterial bioreporter, and, it is much more sensitive than the *zntA*-based *E. coli* bioreporter. The constructed bioreporter is generally less sensitive to Cd than the other bioreporters. Cyanobacterial bioreporters should not be envisaged as an alternative to other microbial bioreporters, but as a useful complement as they inform on pollutant bioavailability/toxicity to organisms at the base of the food webs, namely primary producers. Most authors in the field agree that a battery of bioreporters based on organisms of different trophic levels is a useful tool in environmental monitoring.

Like most of the published microbial bioreporters (Ivask et al., 2002; Magrisso et al., 2008; Hynninen and Virta, 2010), the *Synechococcus* bioreporter responds to several metals. In an environmental sample, it will not discriminate between different ones, but metals as well as other potential toxic pollutants are present in the environment as complex mixtures and antagonistic/synergistic interactions might occur between them (Rodea-Palomares et al., 2010, 2012; Jouanneau et al., 2012). For that reason, this bioreporter might be useful as a first screening tool, which will easily determine whether the sample is polluted with metals, but more specific sophisticated chemical analytical methods are still necessary to determine the chemical species and exact quantities of the metals present. At present, we are evaluating the bioreporter response to heavy metal mixtures of increasing complexity in order to study and define experimental/modeling approaches to understand the potential interactions of these pollutants in the environment.

### Conflict of interest statement

The authors declare that the research was conducted in the absence of any commercial or financial relationships that could be construed as a potential conflict of interest.
