# Supplementary material for: Construction of a self-luminescent cyanobacterial bioreporter that detects a broad range of bioavailable heavy metals in aquatic environments
Source: Front Microbiol. 2015 Mar 9;6:186. doi: 10.3389/fmicb.2015.00186 (PMC4353254; doi:10.3389/fmicb.2015.00186)
Supplement: Supplementary file 1 [file Table1.DOCX]

| Composition | Final Concentration  (µM) |
| --- | --- |
| MgSO_4_ · 7 H_2_O | 200 |
| CaCl_2_ · 2 H_2_O | 240 |
| NaNO_3_ | 18000 |
| K_2_HPO_4_ · 3 H_2_O  EDTA (Tritiplex III) | 230  2.8 |
| Citric acid · H_2_O  Ammonium-iron (III) citrate | 31  20 |
| Na_2_CO_3_ | 190 |
| H_3_BO_3_  MnCl_2_ · 4 H_2_O  ZnSO_4_ · 7 H_2_O*  Na_2_MoO_4_ · 2 H_2_O  CuSO_4_ · 5 H_2_O*  Co(NO_3_)_2_ · 6 H_2_O* | 46  9.1  0.77  1.6  0.32  0.17 |
| MOPS | 2 mM |
| pH = 7.5 |  |

**Table S1**. BG11 medium composition

*: Modified medium lacking Co, Ni and Cu was used for bioluminescence assays.
