# Supplementary material for: Construction of a self-luminescent cyanobacterial bioreporter that detects a broad range of bioavailable heavy metals in aquatic environments
Source: Front Microbiol. 2015 Mar 9;6:186. doi: 10.3389/fmicb.2015.00186 (PMC4353254; doi:10.3389/fmicb.2015.00186)
Supplement: Supplementary file 2 [file Table2.DOCX]

**Table S2**. Predicted percentages of metal present as free ion and other main chemical species as predicted by Visual MINTEQ for inducing and not inducing metals used. The chosen concentration for all metals is 1 µM.

| Inducing metals | Zn | Cd | Ag | Cu | Hg | Co |
| --- | --- | --- | --- | --- | --- | --- |
|  | 18.594 Zn^2+^ 7.889 ZnOH^+^ 0.167 Zn(OH)_2_ AQ 0.240 ZnSO_4_ AQ 1.921 ZnHPO_4_ AQ 0.480 ZnCO_3_ AQ 65.861 ZnCITRATE  4.090 ZnEDTA^2-^ | 50.347 Cd^2+^ 1.370 CdCl^+^ 1.299 CdNO_3_^+^ 0.856 CdSO_4_ AQ  4.132 CdHPO_4_ AQ 0.626 CdHCO_3_^+^ 28.584 CdCITRATE  12.596 CdEDTA^2-^ | 57.422 Ag^+^ 40.055 AgCL AQ 2.003 AgCL_2_^-^ 0.119 AgSO_4_^–^  0.399 AgNO_3_ AQ | 1.052 Cu^2+^ 0.714 CuCO_3_ AQ 0.436 CuOH^+^ 9.923 Cu(OH)_2_ AQ 34.017 CuEDTA^2-^ 53.683 Cu-CITRATE | 99.989 HgEDTA^2-^ | 87.694 Co^2+^  12.306CoEDTA^2-^ |
| Not inducing metals | Pb | Mg | Ni | Fe | Ba | Sr |
|  | 9.786 Pb^2+^ 4.057 PbOH^+^ 1.490 PbNO_3_^+^ 21.977 PbCO_3_ AQ 0.732 PbHCO_3_^+^ 0.611 Pb-CITRATE 60.275 PbEDTA^2-^ | 93.694 Mg^2+^ 0.109 MgHCO_3_^+^ 0.977 MgSO_4_ AQ 0.321 MgPO_4_^-^ 4.129 MgHPO_4_ AQ  0.648 Mg-CITRATE | 2.681 Ni^2+^  2.571 NiCO_3_ AQ 31.7 NICITRATE  62.872 NiEDTA^2-^ | 15.403 Fe^2+^ 58.239 FeOH^+^ 0.231 FeH2PO_4_^+^  3.175 FeHPO_4_ AQ 21.982 FeCITRATE | 96.828 Ba^2+^  3.172 BaCITRATE | 100.000 Sr^2+^ |
