# Supplementary material for: Construction of a self-luminescent cyanobacterial bioreporter that detects a broad range of bioavailable heavy metals in aquatic environments
Source: Front Microbiol. 2015 Mar 9;6:186. doi: 10.3389/fmicb.2015.00186 (PMC4353254; doi:10.3389/fmicb.2015.00186)
Supplement: Supplementary file 3 [file Table3.DOCX]

**Table S3**. Toxicity of heavy metals towards *Synechococcus* sp. PCC 7942 pBG2120 expressed as EC_50_ values (µM) ± standard deviation after 20 h of exposure time

| Metal | *Synechococcus* sp. PCC 7942 pBG2120 |
| --- | --- |
|  | EC_50_ (µM) |
| Zn^2+^ | 1.6±0.15 |
| Cd^2+^ | 2.37±0.56 |
| Ag^+^ | 0.41±0.18 |
|  |  |
| Hg^2+^ | 59.5±5.44 (pM) |
| Cu^2+^ | 0.026±0.003 |
| Co^2+^ | 0.67±0.21 |
