# Supplementary material for: Construction of a self-luminescent cyanobacterial bioreporter that detects a broad range of bioavailable heavy metals in aquatic environments
Source: Front Microbiol. 2015 Mar 9;6:186. doi: 10.3389/fmicb.2015.00186 (PMC4353254; doi:10.3389/fmicb.2015.00186)
Supplement: Supplementary file 4 [file Table4.DOCX]

**Table S4.** Comparison of the performance of *Synechococcus* sp. PCC 7942 pBG2120 with other microbial bioreporter assays

| Promoter | Element | Reporter gene | Host | Linear response (µM) | Time of induction | Medium | Reference |
| --- | --- | --- | --- | --- | --- | --- | --- |
| *smt* | Hg^2+^ | LuxCDABE (*P.luminescens*) | *Synechococcus* | 11-72 (pM) | 240 min | BG-11 | This study |
|  | Cu^2+^ |  |  | 0.027-0.05 |  |  |  |
|  | Ag^+^ |  |  | 0.05-0.29 |  |  |  |
|  | Co^2+^ |  |  | 0.88-2.66 |  |  |  |
|  | Zn^2+^ |  |  | 0.97-2.04 |  |  |  |
|  | Cd^2+^ |  |  | 1.54-5.35 |  |  |  |
| *smt* | Zn^2+^ | *LuxCDABE* (*A.fischeri*) | *Synechococcus* | 0.5-2 | 240 min | BG-11 | ([Erbe et al., 1996](#_ENREF_3)) |
|  | Cd^2+^ |  |  | 0.5-1.5 | 60 min |  |  |
|  | Cu^2+^ |  |  | 2-4 | 120 min |  |  |
| *MTT5* | As^5+^ | *lucFF* (Firefly) | *T.termophila* | 25·10^-3^ (LOD) | 120 min | Tris-HCl | ([Amaro et al., 2011](#_ENREF_1)) |
|  | Zn^2+^ |  |  | 1.5-4.6·10^6^ |  |  |  |
|  | Cd^2+^ |  |  | 5·10^-3^-27 |  |  |  |
|  | Hg^2+^ |  |  | 2.5·10^-2^-7.5 |  |  |  |
|  | Pb^2+^ |  |  | 5·10^-2^-1.5·10^3^ |  |  |  |
|  | Cu^2+^ |  |  | 1.5-2.2·10^3^ |  |  |  |
| *MTT1* | As^5+^ | *lucFF* (Firefly) | *T.termophila* | 50·10^-3^ (LOD) | 120 min | Tris-HCl | ([Amaro et al., 2011](#_ENREF_1)) |
|  | Zn^2+^ |  |  | 0.5-2.29·10^3^ |  |  |  |
|  | Cd^2+^ |  |  | 2.5·10^-2^-8.9 |  |  |  |
|  | Hg^2+^ |  |  | 2.5·10^-2^-5 |  |  |  |
|  | Pb^2+^ |  |  | 0.5-2.4·10^2^ |  |  |  |
|  | Cu^2+^ |  |  | 2.5-7.9·10^2^ |  |  |  |
| *coaT* | Zn^2+^ | *luxAB* (*A.harveyi*) | *Synechocystis* | 1-3 |  | BG-11 | ([Peca et al., 2008](#_ENREF_5)) |
|  | Co^2+^ |  |  | 0.3-6 | 180 min |  |  |
| *copA* | Ag^+^ | *luxCDABE* (*A.fischeri*) | *E.coli* | 0.3-3 | 80 min | GGM minimal | ([Riether et al., 2001](#_ENREF_7)) |
|  | Cu^2+^ |  |  | 3-30 |  |  |  |
| *zntA* | Zn^2+^ | *lucFF* (Firefly) | *E.coli* | 40-15000 | 120 min | M9 | ([Ivask et al., 2002](#_ENREF_4)) |
|  | Cd^2+^ |  |  | 0.05-30 |  |  |  |
|  | Hg^2+^ |  |  | 0.01-1 |  |  |  |
| *merR* | Cd^2+^ | *lucFF*(Firefly) | *P.fluorescens* | 1-10 | 120 min | PBS | ([Petanen et al., 2001](#_ENREF_6)) |
|  | Hg^2+^ |  |  | 10^-5^-0.1 |  |  |  |
| *copA* | Cd^2+^ | *luxCDABE* (*A.fischeri*) | *E.coli* | 0.05 (LOD) |  | Acetate | ([Charrier et al., 2011](#_ENREF_2)) |
|  | Hg^2+^ |  |  | 5 (LOD) |  |  |  |
|  | Ag^+^ |  |  | 0.5 (LOD) |  |  |  |

LOD : limit of detection

Amaro, F., Turkewitz, A.P., Martin-Gonzalez, A., and Gutierrez, J.C. (2011). Whole-cell biosensors for detection of heavy metal ions in environmental samples based on metallothionein promoters from Tetrahymena thermophila. *Microb Biotechnol* 4**,** 513-522.

Charrier, T., Durand, M.J., Jouanneau, S., Dion, M., Pernetti, M., Poncelet, D., and Thouand, G. (2011). A multi-channel bioluminescent bacterial biosensor for the on-line detection of metals and toxicity. Part I: design and optimization of bioluminescent bacterial strains. *Anal Bioanal Chem* 400**,** 1051-1060.

Erbe, J.L., Adams, A.C., Taylor, K.B., and Hall, L.M. (1996). Cyanobacteria carrying an smt-lux transcriptional fusion as biosensors for the detection of heavy metal cations. *J Ind Microbiol* 17**,** 80-83.

Ivask, A., Virta, M., and Kahru, A. (2002). Construction and use of specific luminescent recombinant bacterial sensors for the assessment of bioavailable fraction of cadmium, zinc, mercury and chromium in the soil. *Soil Biology and Biochemistry* 34**,** 1439-1447.

Peca, L., Kos, P.B., Mate, Z., Farsang, A., and Vass, I. (2008). Construction of bioluminescent cyanobacterial reporter strains for detection of nickel, cobalt and zinc. *FEMS Microbiol Lett* 289**,** 258-264.

Petanen, T., Virta, M., Karp, M., and Romantschuk, M. (2001). Construction and Use of Broad Host Range Mercury and Arsenite Sensor Plasmids in the Soil Bacterium Pseudomonas fluorescens OS8. *Microb Ecol* 41**,** 360-368.

Riether, K.B., Dollard, M.A., and Billard, P. (2001). Assessment of heavy metal bioavailability using Escherichia coli zntAp::lux and copAp::lux-based biosensors. *Appl Microbiol Biotechnol* 57**,** 712-716.
