# Supplementary figures and images for: Construction of a self-luminescent cyanobacterial bioreporter that detects a broad range of bioavailable heavy metals in aquatic environments
Source: Front Microbiol. 2015 Mar 9;6:186. doi: 10.3389/fmicb.2015.00186 (PMC4353254; doi:10.3389/fmicb.2015.00186)

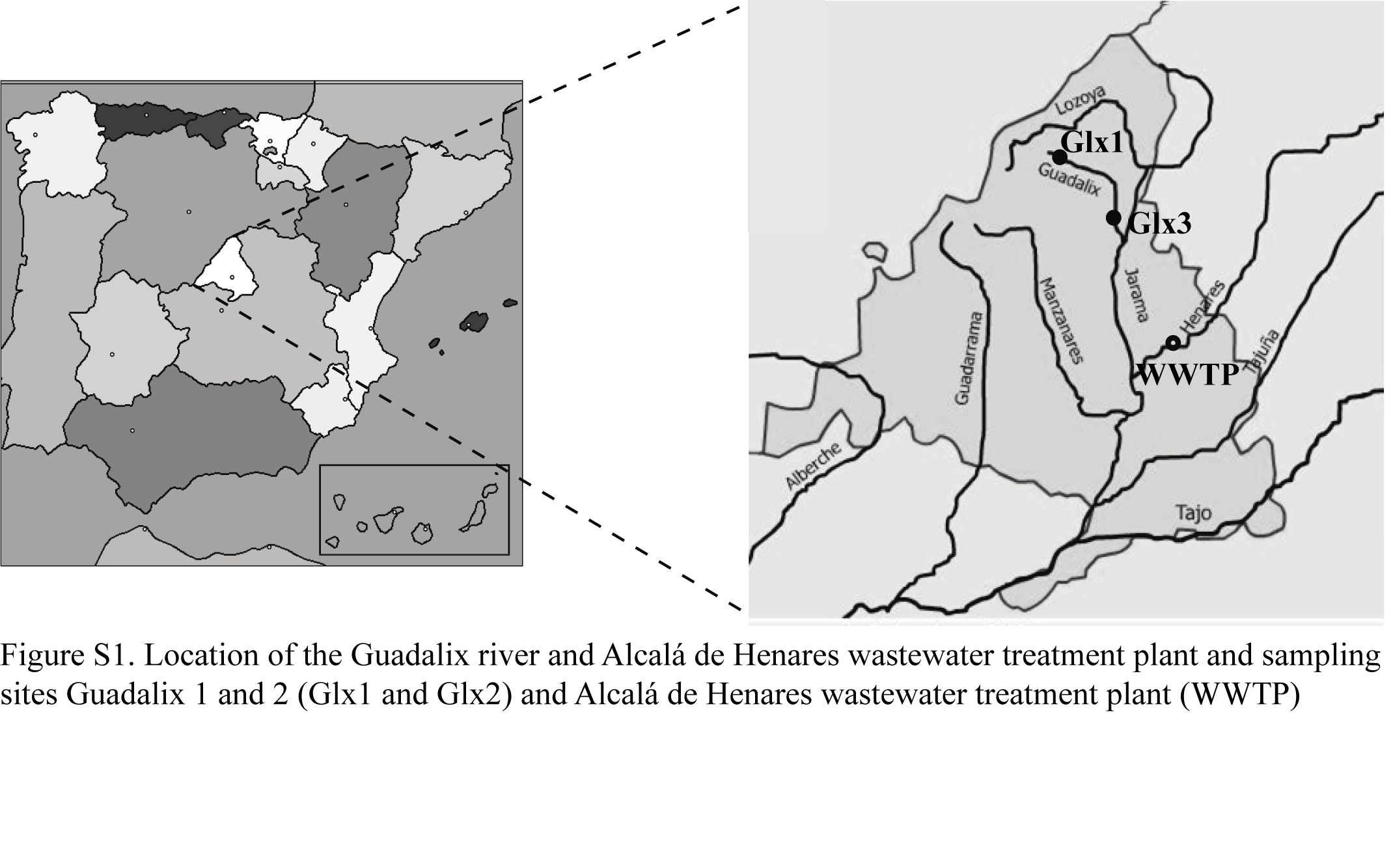

Supplement: Supplementary file 5 [file Image1.TIF]

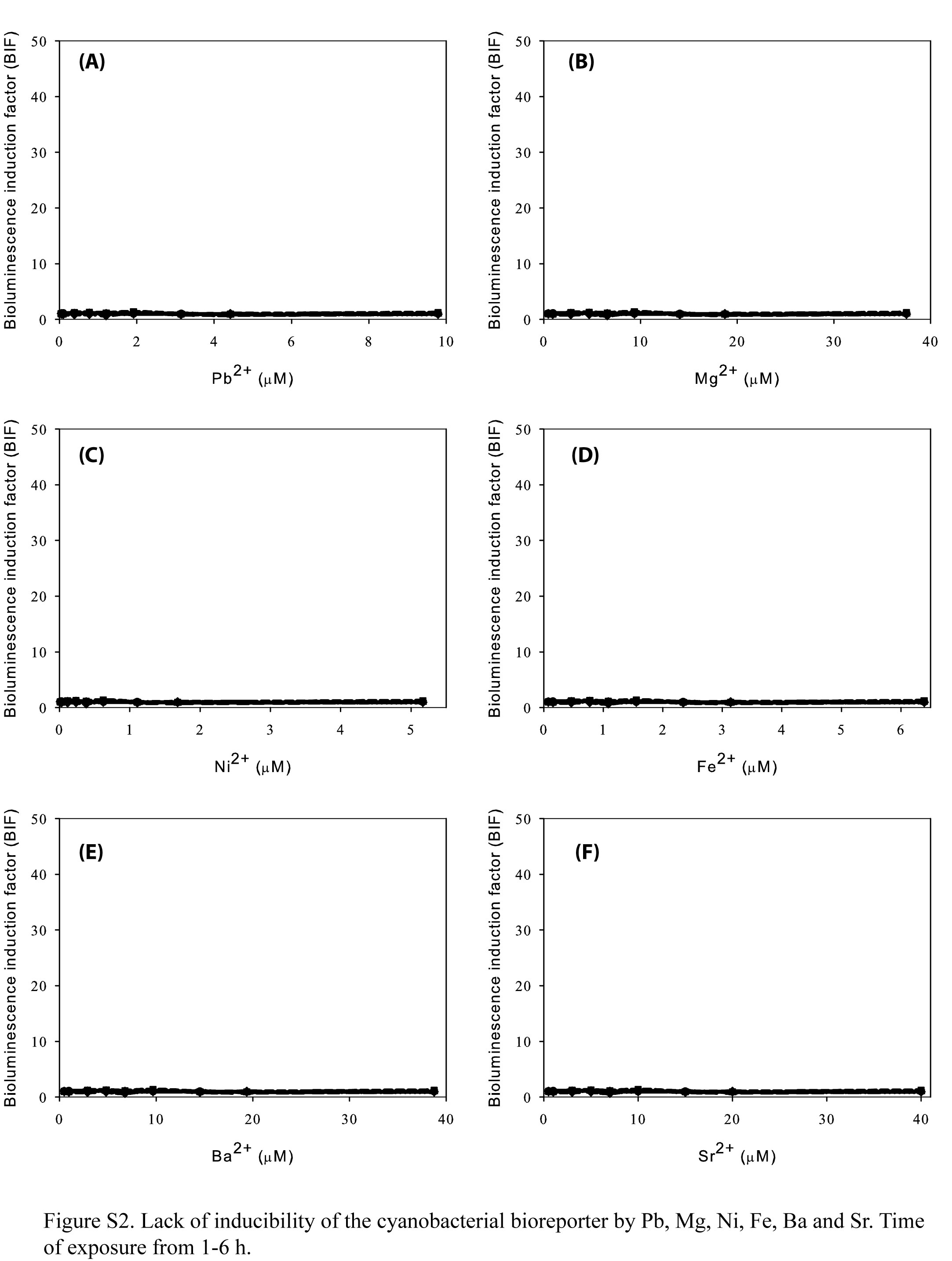

Supplement: Supplementary file 6 [file Image2.JPEG]
